# Magnetic resonance imaging assessment of myocardial inflammation in 132 unselected, consecutive patients with clinical suspicion of acute or chronic myocarditis - are we as good as we thought?

**DOI:** 10.1186/1532-429X-13-S1-O3

**Published:** 2011-02-02

**Authors:** Philipp Lurz, Julia Murawski, Julia Steiner, Mahdi Sareban, Ingo Eitel, Steffen Desch, Georg Fuernau, Suzanne de Waha, Matthias Grothoff, Christian Luecke, Stephan Nitzsche, Gerhard Schuler, Matthias Gutberlet, Holger Thiele

**Affiliations:** 1 University of Leipzig, Heart Centre Leipzig, Germany, Leipzig, Germany

## Background

Endomyocardial biopsy (EMB) represents the gold-standard for the assessment of myocardial inflammation in the context of suspected myocarditis. Recently, several studies have reported an encouraging diagnostic performance of cardiac magnetic resonance imaging (MRI) for the assessment of myocardial inflammation/myocarditis. However, the comparison of MRI results to clinical data only and the use of preselected patient populations are important limitations of the majority of these reports.

## Objective

We sought to define the diagnostic performance of MRI for the assessment of myocardial inflammation and necrosis as compared to EMB in an unselected cohort of consecutive patients with suspected acute and chronic myocarditis.

## Methods

132 consecutive patients with suspected acute or chronic myocarditis were included in this prospective trial. Patients had to fulfill indications for CMR imaging in suspected myocarditis according to a published consensus report (“JACC White Paper”).

All patients underwent left ventricular EMB, cardiac catheterization for exclusion of coronary artery disease, and MRI on a 1.5 Tesla scanner (Intera, CV, Philips Medical Systems). Imaging protocols included T2-weighted imaging for calculation of the edema ratio (ER), T1-weighted imaging before and after contrast agent administration for calculation of the global relative enhancement (gRE) and assessment of late enhancement (LE) for detection of myocardial necrosis, scarring or fibrosis. Assessment and interpretation ER, gRE, and LE were performed according to the JACC White myocarditis consensus paper. MRI imaging results were considered to be consistent with the diagnosis of myocarditis if 2 out of 3 (ER, gRE, LE) of the MRI criteria were positive.

Patients were divided into 2 groups according to duration of symptoms: patients with presumed acute myocarditis (symptoms equal to or < 14 days) and those with presumed chronic myocarditis (symptoms > 14 days).

## Results

The diagnostic performance including sensitivity, specificity and accuracy for the individual imaging techniques is summarized in Table 1. In all patients, the accuracy of our comprehensive MRI protocol for assessment of myocarditis as compared to EMB was 66%. Further, accuracy of MRI was clearly enhanced when performed in patients with suspected acute myocarditis as compared to patients with suspected chronic myocarditis (accuracy 77 vs. 52%, respectively).

**Table 1:** Diagnostic performance of MRI for the assessment of myocardial inflammation in an unselected cohort of consecutive patients (a), in patients with suspected acute myocarditis (b) and in patients with suspected chronic myocarditis (c). 2/3, 2 out of 3 MRI criteria (ER, gRE, LE) positive.

	ER	gRE	LE	2/3
**(a) All patients, n=132**				
Sensitivity (%)	52	74	68	74
Specificity (%)	65	31	44	53
Accuracy (%)	57	58	59	66
**(b) Symptoms ≤ 14 days, n=70**				
Sensitivity (%)	58	75	73	81
Specificity (%)	61	50	61	67
Accuracy (%)	59	69	70	77
**(c) Symptoms > 14 days, n=63**				
Sensitivity (%)	44	72	59	63
Specificity (%)	67	20	33	40
Accuracy (%)	55	47	47	52

## Conclusion

The results of our study underline the usefulness of MRI for assessment of myocardial inflammation/myocarditis in patients with suspected acute myocarditis. In contrast, with the current criteria, techniques and sequences, the diagnostic performance of MRI in patients with suspected chronic myocardial inflammation/myocarditis might be not sufficient to guide clinical management.

